# Proximal Upper Limb Sensorimotor Integration in Response to Novel Motor Skill Acquisition

**DOI:** 10.3390/brainsci10090581

**Published:** 2020-08-22

**Authors:** Sinead O’Brien, Danielle Andrew, Mahboobeh Zabihhosseinian, Paul Yielder, Bernadette Murphy

**Affiliations:** Faculty of Health Sciences, University of Ontario Institute of Technology, 2000 Simcoe St. North, Oshawa, ON L1H 7K4, Canada; sinead.obrien@ontariotechu.ca (S.O.); danielle.andrew@ontariotechu.ca (D.A.); mahboobeh.zabihhosseinian@ontariotechu.ca (M.Z.); paul.yielder@ontariotechu.ca (P.Y.)

**Keywords:** somatosensory evoked potentials, SEPs, motor acquisition, upper limb

## Abstract

Previous studies have shown significant changes in cortical and subcortical evoked potential activity levels in response to motor training with the distal upper-limb muscles. However, no studies to date have assessed the neurological processing changes in somatosensory evoked potentials (SEPs) associated with motor training whole-arm movements utilizing proximal upper-limb muscles. The proximal upper-limb muscles are a common source of work-related injuries, due to repetitive glenohumeral movements. Measuring neurophysiological changes following performance of a proximal motor task provide insight into potential neurophysiological changes associated with occupational postures and movements involving proximal upper limb muscles. This study sought to assess the impact of a novel motor skill acquisition task on neural processing of the proximal upper-limb muscle groups, through the measurement of short-latency median nerve SEPs. One group of 12 participants completed a novel motor training task, consisting of tracing a sinusoidal waveform varying in amplitude and frequency. Baseline SEP measurements were recorded from each participant, followed by a mental recitation control task. Pre-test SEP measurements were then recorded, followed by the motor training task, and post-test SEP recordings. The participants completed the tracing with their right thumb, using glenohumeral rotation only to move their hand. Significant improvements in task accuracy were demonstrated, indicating that motor acquisition had occurred. Significant changes were also seen in the N11, N13, N20, N24, P25, and the N30 SEP peaks were seen following the motor training task. Conclusion: Early SEPs appear to be a sensitive measure of changes in sensorimotor integration in response to novel motor skill acquisition within the proximal upper-limb muscles.

## 1. Introduction

A key aspect of learning is the ability of the brain to adaptively change the way it processes incoming information based on past experiences. This ability to adapt is referred to as cortical plasticity and underlies our ability to learn new skills and recover from injury [1]. Learning a skill requires training and repetition; in some instances, there can be deteriorations in movement due to fatigue or even pain as a result of prolonged overuse. In overuse conditions, studies have indicated that dysfunctional cortical plasticity may play an important role in motor degradation [2,3]. Overuse injuries have become prominent in the workplace, with musculoskeletal disorders being one of the most common self-reported illnesses. Among musculoskeletal disorders, upper limb disorders rank extremely high in prevalence due to repetitive motions, abnormal pressures and excessive forces [4,5]. As a spheroid joint, the shoulder is the most mobile joint which integrates the various structures surrounding the shoulder and upper limb [6]. The activation of major muscle groups of the trunk requires range of motion from the shoulder complex, implicating its importance in various daily tasks.

The shoulder has evolved to withstand heavy demands over a wide range of motion. However, the complexity of its structure makes it susceptible to a range of pathologies which contribute to this increased prevalence observed in upper limb musculoskeletal disorders [7]. Examples of these injuries include rotator cuff impingement, tendinitis, and adhesive capsulitis [7]. 

Various studies have sought to understand the mechanisms of plasticity in response to motor training within the upper limb, although many have been focused on distal muscles within the hand [8,9,10]. One of the oldest principles of motor learning is the specificity of practice hypothesis, which posits that training effects are highly task and effector specific [11,12,13]. Studies have shown that learning effects are highly specific to the particular task trained [14], making it important to study proximal upper limb muscle groups given the unique susceptibility of the glenohumeral joint to injury. Further evidence for understanding the mechanisms of motor acquisition for proximal upper limb muscles is explained by studies which have demonstrated that there is an asymmetrical transfer of acquisition effects between proximal and distal effectors such that the generalizability of acquisition is larger for proximal effectors compared to distal ones [14]. This could be critical when developing rehabilitative strategies for individuals recovering from musculoskeletal injuries or stroke. 

The development of a whole-arm motor acquisition task would allow for better understanding of neuroplastic changes associated with the types of movements that occur in workplace tasks requiring repetitive shoulder movements. Short-latency somatosensory evoked potentials (SEPs) (<30 ms) are potentials measured over the scalp, and their amplitude reflects the activity of neural generators in response to sensory stimulation, often elicited experimentally via median nerve stimulation. Early SEP amplitude changes have been found in response to motor skill acquisition involving the distal hand muscles [9,10,15]. The current study built on the motor skill acquisition tracing task utilized by Andrew et al. (2015) [8]. The task was adapted to use with the whole arm, and required the use of proximal upper-limb muscles to perform the task. The aim of this study was to determine whether short-latency SEPs changed in response to a proximal upper-limb motor acquisition-tracing task, which would validate the use of median nerve SEPs to measure changes in cortical plasticity for future work involving proximal upper limb muscles. 

## 2. Materials and Methods

### 2.1. Inclusion and Exclusion Criteria

The technique used within this study to assess SEPs relies on the full functionality of the nervous system; therefore, any neurological deficits (demyelination, cerebellar lesions, etc.) would interfere with SEPs and thus were excluded. Classically trained musicians have displayed functional and structural changes in both sensorimotor and auditory cortical systems, which could potentially impact any motor acquisition resulting from the tracing task and were therefore excluded [16]. As this study aimed to compare upper-limb motor acquisition tasks, right hand-dominant subjects were selected to reduce the potential influence of confounding factors resulting from differences in motor acquisition strategies between the two arms [17]. Handedness was confirmed by Edinburgh Handedness Inventory (EHI) [18]. Throughout each data collection session, participants were seated in a comfortable, upright chair with armrests and were instructed to remain as quiet and still as possible throughout the testing session. During all SEP recordings, the lights in the room were turned off to minimize electrical noise, and participants were asked to keep their eyes open.

### 2.2. Participants

12 right-handed participants (6 males and 6 females), aged 19–25 (mean age 20.25, ±1.82 years), participated. All participants consented to two experimental sessions: a motor acquisition intervention, and a retention test no less than 24 h and no more than 48 h following the initial session. Each participant provided informed consent and completed a pre-screening safety questionnaire. This study received ethical approval by the University of Ontario Institute of Technology Research Ethics Board. Each participant completed an informed consent form prior to participation in the study. This study was carried out according to the ethical standards set out by the Declaration of Helsinki for the use of humans in experimental studies.

The use of SEP peak recordings was chosen to measure cortical and subcortical activity changes during this study due to its successful use in previous, similar studies [8,9,10,19,20]. Previous studies have used fine motor tasks performed by the distal hand muscles. This study aimed to validate the use of SEPs to assess neurophysiological changes in response to the performance of whole-arm tasks, and their ability to potentially investigate neuroplastic changes in proximal upper limb muscles.

### 2.3. Stimulation of the Median Nerve

The stimuli delivered to each participant consisted of 1500 electrical square pulses of 1 ms in duration, with an optically isolated Digitimer (DSA-7). The stimulus was delivered through Ag/AgCl ECG conductive adhesive electrodes (MEDITRACE™ 130 by Ludlow Technical Products Canada Ltd., Mansfield, MA, USA) (impedance ≤ 5 Ω) placed over the median nerve at the wrist of the right hand between the tendons of flexor pollicis longus and palmaris longus. SEPs were delivered at rates of both 2.47 Hz and 4.98 Hz. The faster rate allowed for the attenuation of the N30 SEP peak, and therefore, accurate measurement of the N24 peak. The slower rate does not result in SEP peak attenuation, and allowed for the accurate measurement of the remaining peaks [10,21]. The stimulus intensity was increased for each participant until the first visible muscle contraction of the abductor pollicis brevis muscle.

To minimize the electrical artifacts, a ground electrode (1.8288 m Traditional Lead, 10 mm disc, 2 mm hole gold cup electroencephalography (EEG) electrode) was placed in the mouth of each participant. The C5 spinous process was referenced to the trachea, while Erb’s point and the cephalic site electrodes were referenced to the ipsilateral earlobe. 

### 2.4. SEPS Recording Parameters

The recording surface electromyography (EMG) electrodes were placed as follows: (a) on the ipsilateral Erb’s point (brachial plexus) for upper extremity SEPs; (b) over the C5 spinous process, on the anterior side of the trachea. The cephalic site recording EEG electrodes were placed on (c) a parietal site (CC’) 2 cm posterior to contralateral central C3/4; and (d) a frontal site (Rossi site) 6 cm anterior and 2 cm contralateral to vertex or Cz [22]. 

The amplitudes and latencies of the following short-latency SEP components were identified and analyzed: (a) the peripheral N9; (b) the spinal N13; (c) the far-field N18; (d) the parietal N20, and P25; and (e) the frontal N24 and N30.

### 2.5. Data Collection

The SEP recording was composed of 1500 electrical stimulations (sweeps), which were delivered at the chosen frequency, through a Signal^®^ configuration (Cambridge Electronic Design, Cambridge, UK). The SEP signal was amplified (gain: 10,000), filtered between 0.2–1000 Hz, and then stored on the laboratory computer. The recording was then averaged over all 1500 sweeps for each stimulation rate. 

### 2.6. Motor Acquisition Tracing Task Design

**Figure 1:**
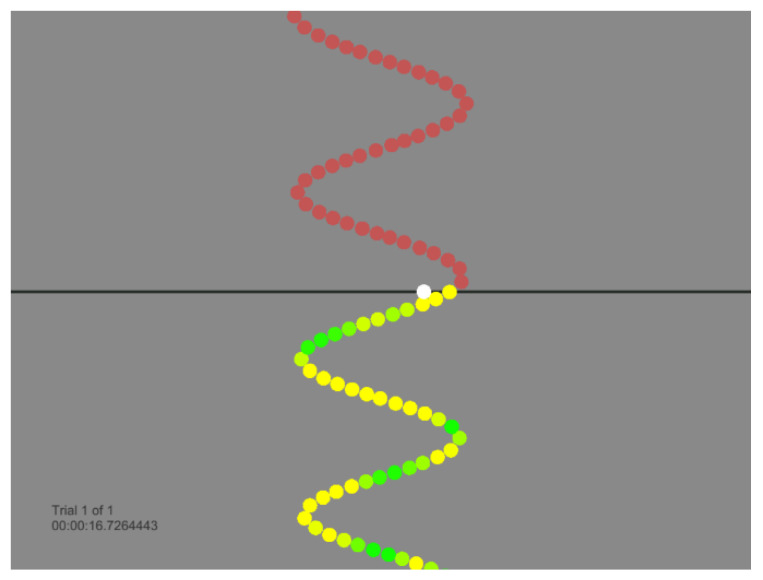
Motor acquisition tracing task design: participants attempted to trace a scrolling sinusoidal wave form composed of dots, using their right thumb. Continuous sinusoidal pattern waves moved vertically down the screen and participants attempted to copy each dot as it passed the horizontal line. The colour of the dots that comprised the sinusoidal wave ranged from green to represent good accuracy, and yellow to representing less accurate performance.

The motor skill acquisition-tracing task used within this study was developed with the Unity™ (Personal Edition) game development software (Unity Technologies, San Francisco, CA, USA). The task required participants to trace scrolling sinusoidal wave forms composed of dots, using their right thumb. Continuous sinusoidal pattern waves moved vertically down a monitor and participants attempted to trace each dot. To allow for feedback and in-task error correction, the colour of the dots that comprised the sinusoidal wave ranged from green (perfect accuracy, 0% error) to varying shades of yellow (>0% error), as displayed in Figure 1. The sinusoidal wave included four patterns of varying amplitude and frequency, to provide high levels of contextual interference. Contextual interference refers to the effect in which interference during task practice leads to superior task performance and retention. Thus, high levels of contextual interference will result in the optimal learning environment within a motor learning paradigm [23]. Each trial consisted of 500 dots. For the pre- and post-tests, each of the four patterns was performed once; for the acquisition phase, each version was performed three times for a total of 12 traces. During each trial, accuracy was measured in dot widths for the distance from the target trace. One dot width away from the target trace was equal to 100% error [8,9,15,24]. 

### 2.7. Motor Acquisition Task Intervention

The sinusoidal waves were displayed on the first monitor (laptop) on the table, and participants attempted to trace the dots on the second touchscreen monitor that was placed on a desk directly below the table, ensuring that the table blocked the participants’ view of their own arm so that they could not see the movement of their right thumb and hand during the tracing trials. To control the sweep of each sinusoidal wave, participants were required to adjust to the velocity and amplitude of the traces by performing internal and external rotation of the right shoulder joint.

**Figure 2:**
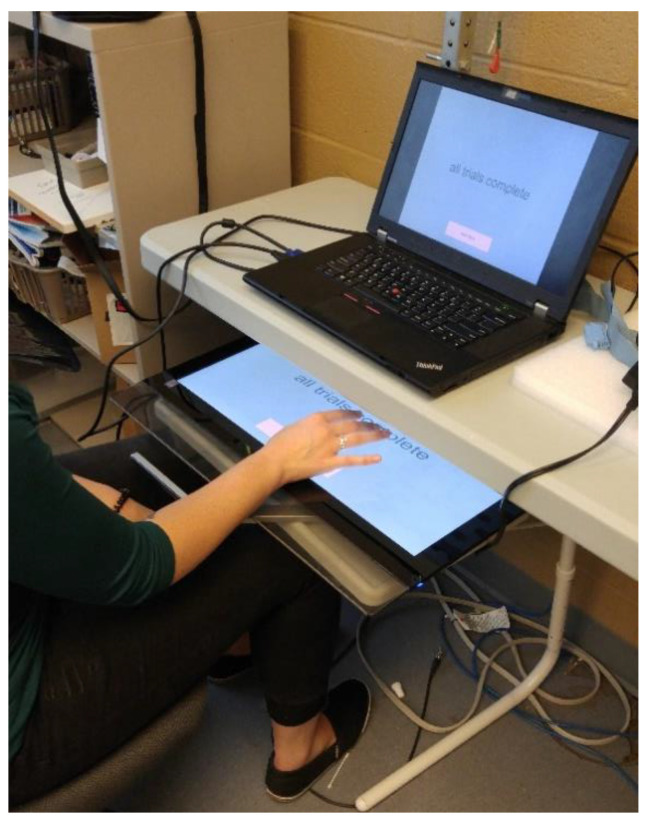
Motor skill acquisition tracing task setup. A laptop is placed on the table, displaying the motor training task. A large touchscreen is placed on a desk directly below the table, ensuring that the table blocks the participants’ view of their own arm. To control the sweep of each sinusoidal wave, participants were required to adjust to the velocity and amplitude of the traces by performing internal and external rotation of the right shoulder joint.

Participants were instructed to hold their elbow, wrist, and hand rigid, while using glenohumeral internal and external rotation to move their hand across the second touchscreen monitor spatial and temporal patterns of thumb and shoulder movement, as displayed in Figure 2. This was closely monitored by the experimenter to ensure that participants were isolating only movement of the glenohumeral joint to complete the task throughout the duration of the study.

**Figure 3:**
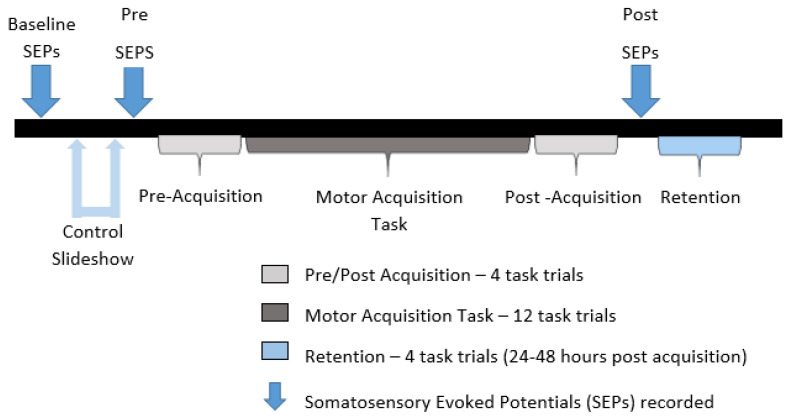
Experimental procedure.

For each of the participants within the study, baseline SEP measurements were recorded before the pre-test trials. This was followed by a mental recitation task, in which participants mentally recited sequences of numbers on a slide presentation (PowerPoint). This mental recitation task was included to control for the potential effects of attention on SEPs and to ensure that any potential changes seen following the tracing task were in fact motor related. Pre-test SEP measurements were then performed, followed by the motor skill acquisition task. Behavioural measures of accuracy were recorded during the pre- and post-test phases of the task. Lastly, post-test SEP measurements were taken. After a minimum of 24 h and a maximum of 48 h, dependent upon participant availability, participants were required to return for a retention test, during which four trials of the tracing task were performed and accuracy was assessed, as outlined in Figure 3.

### 2.8. Data Analysis

Changes in SEP peak amplitude and latency were measured at baseline, following the control intervention, prior to motor skill acquisition, and post-acquisition. Additionally, task accuracy was measured during the first four acquisition trials, the last four acquisition trials, and during the retention session. In order to make changes in SEP peak magnitude comparable between subjects, all SEP data were normalized to each participant’s baseline value, and expressed as a proportion of the baseline measurement. A repeated measures ANOVA was performed in SPSS (IBM Software), with pre-planned comparisons to baseline, for both the post-control and post-acquisition SEP amplitudes to the initial baseline amplitude. Statistical significance was set at *p* < 0.05. To ensure stable peripheral nerve volleys, only participants with a stable N9 recording (e.g., within ±10% of the baseline N9 value) were included in statistical analysis. As the N9 records the arrival of the peripheral nerve volley to the brachial plexus, trials in which the N9 differs by greater than ±10% could indicate alterations in the incoming afferent volley, possibly due to postural changes. The amplitude of each SEP peak was measured from peak of interest to the preceding or succeeding peak or trough, in accordance with international recommendations [25,26]. Amplitudes and latencies were measured for the following peaks: N9, N11, N13, P14, N18, N20, N24, P25, and N30. 

## 3. Results

All 12 participants who completed the study were included in the statistical analysis of SEP amplitudes and in the statistical analysis of the task accuracy data. 

### 3.1. Neurophysiological Data

**Figure 4:**
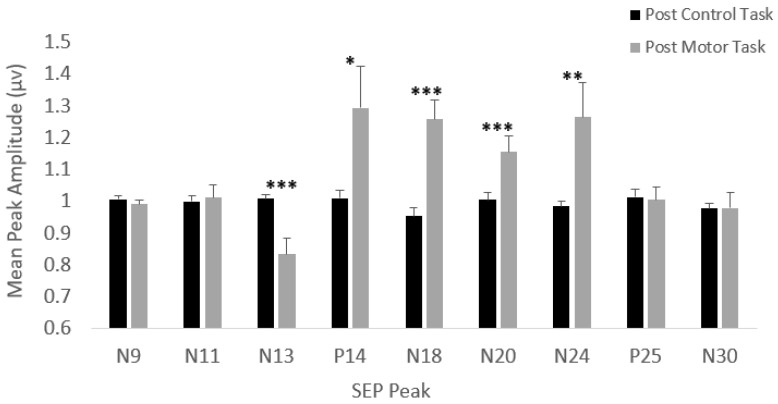
Normalized mean percent amplitude change following control task and motor training task, +SE. Note the significant change in post-motor task amplitude for the N13, P14, N18, N20, and N24 peaks. * *p* < 0.05, ** *p* < 0.01, *** *p* < 0.001, with respect to baseline. Normalized baseline somatosensory evoked potential (SEP) amplitudes are equal to one.

**Figure 5:**
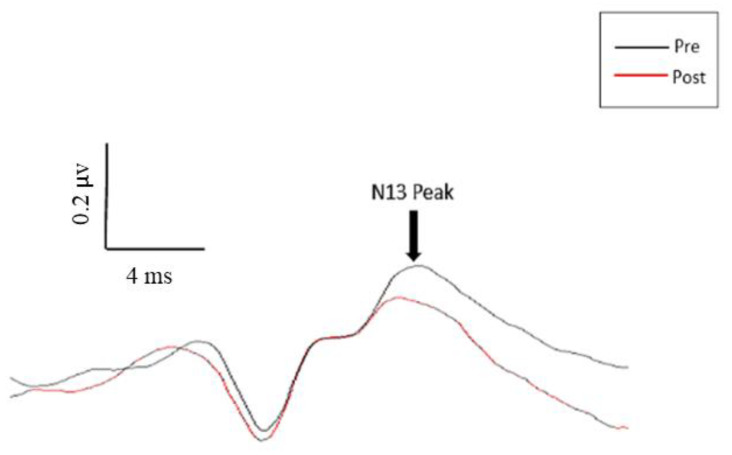
Pre- and post-tracing intervention SEP peaks for one representative participant. The black trace is the pre-intervention trace; the red trace is the post-intervention trace. Note the large decrease in amplitude for the N13 peak.

**Figure 6:**
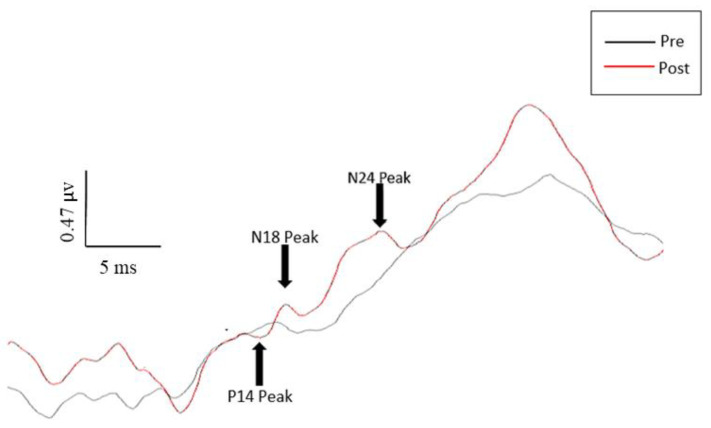
Pre- and post-tracing intervention SEP peaks for one representative participant. Black trace is the pre-intervention trace; red trace is the post-intervention trace. Note the large increases in amplitude for the P14, N18, and N24 peaks.

No significant changes in latency were seen for any of the measured peaks. As displayed in Figure 4, significant changes in amplitude were seen for the N13, P14, N18, N20, and N24 SEP peaks following motor acquisition. No significant changes in amplitude were seen following the control task, as displayed in Figure 4. This is further displayed in the representative traces pictured in Figure 5 and Figure 6.

#### 3.1.1. N9 SEP Peak (Erb’s Point)

For all but one participant (for the faster rate SEP recordings), the N9 met the criteria of being within ±10% for all recordings. Additionally, the repeated measures ANOVA indicated that there were no significant differences in the N9 SEP peak between baseline, control, and post-intervention amplitudes. This indicates that any succeeding changes in SEP amplitudes resulted from changes in neural processing, rather than altered afferent input. 

#### 3.1.2. N13 SEP Peak (C5 Spinous Process)

The N13 SEP peak showed significant changes following motor training, (F(2,22) = 11.66, *p* < 0.0001), specifically between baseline and post-intervention SEP measures (F(1,11) = 10.56, *p* < 0.01). There was a mean decrease of 16.7% following the motor training task.

#### 3.1.3. P14 SEP Peak (Rossi Site)

Significant increases (29.4%) were seen in the P14 SEP peak following motor training (F(2,22) = 4.91, *p* < 0.05), specifically between baseline and post-intervention (F(1,11) = 5.22, *p* < 0.05).

#### 3.1.4. N18 SEP Peak (Rossi Site)

Following the motor training session, there were significant increases (25.7%) in the N18 SEP peak, (F(2,22) = 19.04, *p* < 0.0001), specifically significant between baseline and post-intervention (F(1,11) = 18.47, *p* = 0.001).

#### 3.1.5. N20 SEP Peak (Cc’)

The N20 SEP peak showed significant increases (15.6%) following motor training (F(2,22) = 8.89, *p* = 0.001), specifically between baseline and post-intervention (F(1,11) = 10.56, *p* < 0.01).

#### 3.1.6. N24 SEP Peak (Rossi Site)

The N24 was recorded separately, at a stimulation rate 4.98 Hz, as the faster rate allowed for the attenuation of the N30 SEP peak, and therefore, accurate measurement of the N24 peak. The data of one participant had to be excluded from analysis of the N24 SEP peak, as the N9 amplitude for that participant did not meet the data inclusion criteria (not varying by more than ±10%) described in the analysis section above. Following the motor training session, the N24 peak displayed significant mean increases of 26.5% (F(2,20) = 6.62, *p* < 0.01), specifically between baseline and post-test (F(1,10) = 6.14, *p* < 0.05).

### 3.2. Behavioural Data

For the task accuracy behavioural data, significant improvements in performance were seen (F(2,22) = 42.15, *p* < 0.0001), specifically from baseline to post-acquisition (F(1,11) = 37.40, *p* < 0.0001), with a normalized mean decrease in error of 36.7%. These improvements in performance were then retained up to 48 h later, with significant improvements in performance from baseline to retention (F(1,11) = 58.68, *p* < 0.0001), a normalized mean decrease in error of 39.6%.

An interesting point to note was that, although there were insufficient numbers to run a between sex analysis, male participants performed better at baseline than their female counterparts, with an average baseline error of 96.3% for males as compared to 119.0% for females, as displayed in Table 1. Relative to baseline, females improved by 37.9% following acquisition, and 44.6% at retention. In contrast, males improved by 30.7% following acquisition, and 35.6% at retention. Based upon the twelve participants within this study (six males and six females), it appears that males performed better at the task at baseline.

## 4. Discussion

There are currently no proximal upper-limb motor acquisition tasks that have been shown to induce significant changes in SEP peaks. This study was the first of its kind to pilot a whole-arm motor acquisition task that lead to changes in SEP peak amplitudes. The observed improvements in accuracy following the acquisition task and retained 24–48 h later further support the changes seen within the cortical peaks immediately following acquisition. Differential SEP changes in spinal compared to cortical peaks may be representative of differences in sensory feedback that is provided to the proximal upper limb muscles. Furthermore, increases in cortical larger sensorimotor network peaks are indicative of skill acquisition, as indexed by the increases in behavioural performance. 

### 4.1. Neurophysiological Measures

#### 4.1.1. N13 SEP Peak

The spinal N13 SEP peak reflects the activity of interneurons within the mid cervical cord and dorsal horn, and is currently thought to reflect changes in sensorimotor integration at the level of the spinal cord [9,25,27]. Previous motor learning studies have displayed significant increases in the N13 SEP peak following a period of motor acquisition with the thumb, indicating greater neural processing at the level of the spinal cord, and potentially reflecting the fact that the thenar muscles have their afferent input directly conveyed by the median nerve [9]. However, significant decreases in the spinal N13 SEP peak within the present study suggest differing neural activations within the spinal cord for the proximal upper-limb muscles. Decreases to the N13 amplitude for the proximal upper-limb muscles could reflect the fact that the sensory feedback from the shoulder muscles is conveyed primarily by the axillary nerve, rather than the median nerve. This could also reflect differences in descending inhibition to the motor neuron pools required to control the shoulder-movement guided tracing task compared to the thumb-movement guided tracing task. In the thumb task, the thumb is the prime mover, whereas, in the shoulder tracing task, the thumb has to be held rigid as the shoulder internal and external rotator muscles become the prime movers.

#### 4.1.2. P14 SEP Peak

The P14 SEP peak is generated at or above the level of the foramen magnum, but below the cortex [25,27], and is thought to be generated by the arrival of the afferent volley at the medial lemniscus, and the nucleus cuneatus within the medulla oblongata [27,28]. The increase in the P14 in this study compared to the absence of changes when completing motor acquisition tasks with the thumb or first finger could be due to higher levels of proprioceptive input processed during the proximal upper-limb task, which required multi-joint control of the entire upper limb, rather than just the thenar muscles.

#### 4.1.3. N18 SEP Peak

Previous work has demonstrated large decreases in the N18 peak following a simple repetitive motor acquisition task [8,10]. Several studies have suggested that the N18 peak reflects inhibitory activity at the level of the medulla and dorsal column nuclei, potentially displaying a level of afferent processing within the brainstem [10,22,29]. Therefore, a decrease in the amplitude of the N18 peak following motor acquisition may suggest a reduction in inhibitory activity, potentially at the level of the cuneate nucleus or the inferior olives [27,29,30,31]. However, the present study using the proximal upper-limb muscles displayed large increases in the N18 peak following motor acquisition. This could reflect the increased inhibition needed to control the thenar and rotator cuff muscles during whole-arm motor acquisition tasks. 

#### 4.1.4. N20 SEP Peak

This increased need for the processing of incoming stimuli is further demonstrated through large increases in the amplitude of the N20 peak. Representing the early cortical processing activity of Brodmann’s area 3b, within the primary somatosensory cortex, the N20 peak displays the role of the somatosensory cortex in motor acquisition [27,29,32,33]. Large increases in the amplitude of the N20 peak following motor training with the proximal upper limb indicate an increased activation of the somatosensory cortex following motor acquisition, in accordance with previous research [9,10].

#### 4.1.5. N24 SEP Peak 

Previous research using patients with unilateral cerebellar lesions has displayed alterations to the amplitude of the N24 peak, suggesting that the N24 peak represents activity within a neuronal pathway linking the cerebellum and the primary somatosensory cortex [22,25,34,35,36]. The large increases in the N24 peak seen with the proximal upper limb further support previous research suggesting the role of the cerebellum in early motor acquisition [10,37,38]. The increase in the N24 could also indicate an increase in cerebellar inhibition to the thenar muscles, due to the fact that the shoulder muscles were the prime mover for this complex motor acquisition task, requiring multi-joint control of the upper limb.

#### 4.1.6. N30 SEP Peak

These differences in sensorimotor integration between distal and proximal muscles are further demonstrated through a lack of changes in the N30 SEP peak for the shoulder tracing task. The N30 peak is a complex loop with multiple neural generators, linking the premotor and motor areas, the thalamus, and the basal ganglia, which reflects changes in sensorimotor integration within the cortex [28,36,39,40]. Previous research has shown changes in the N30 SEP peak following complex motor acquisition tasks performed with the thumb muscle [8,9,15]. The lack of change for the proximal shoulder muscles may reflect that processing was not significantly altered in muscles that were not the prime mover for the task. Interestingly, previous work found increased N30 amplitudes in the median nerve N30 when the radial nerve was transiently deafferented with an anesthetic block. The authors suggested that this was due to the unmasking of latent cortico-cortical and/or thalamo-cortical connections. In the current study, the proximal shoulder muscles involved in the task used, primarily, internal and external shoulder rotation, innervated from the C5-6 spinal level through the axillary nerve (deltoid and teres minor), suprascapular nerve (infraspinatus) and lower subscapular (teres minor). As a complex sensorimotor integration peak with more than one neural generator, there may have been increased processing in some neural generators with decreased processing in others, which our single site N30 recording was unable to capture. Future work using whole-head EEG and source localization software may be able to tease this apart.

### 4.2. Behavioural Measures

**Figure 7:**
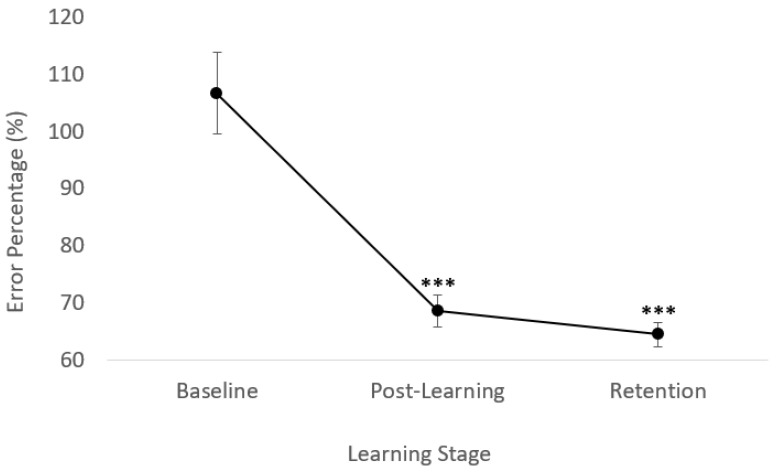
Improvements in tracing accuracy ± SE. *** *p* < 0.0001 relative to baseline.

A control task was included within this study to ensure that any neurological processing changes resulted directly from the motor acquisition task. Following the control task, no significant changes were seen in any SEP amplitudes. Following the acquisition phase of the motor task, the in-task measures of accuracy revealed that overall, there was a significant 36.7% decrease in error, as displayed in Figure 7 above. In addition, these improvements in task performance were retained between 24 to 48 h following acquisition. 

Male and female participants were counterbalanced in this study, based on normative Pegboard data, which suggests sex-based differences in fine motor control [41]. However, additional studies have suggested that these sex-based differences in fine motor control disappear when finger size is used as a covariate [42]. While we lacked the numbers to statistically compare males and females, generally, the females were worse at baseline, with greater rates of improvement. These initial larger improvements in female performance relative to baseline could reflect a potential power law effect, in that relatively larger performance improvements are seen following a worse baseline performance [43]. Future research should assess whether these differences are sex based or performance based, using a motor acquisition task where finger size will not affect task performance.

Significant, relatively permanent improvements in task accuracy within this study have successfully demonstrated motor acquisition within the proximal upper-limb muscles, following a period of motor training. In addition, this work has expanded on previous research that displayed large changes in SEP amplitudes following the same motor acquisition task, using thenar adduction and abduction [9]. While previous studies had used SEPs to show neurological processing changes associated with motor acquisition in the distal hand muscles, this study is the first of its kind to measure SEPs in conjunction with a proximal upper-limb motor skill task.

Understanding the changes that occur during skill acquisition with proximal upper limb muscles is critical, especially given the differential changes that are observed as compared to studies which have looked at distal upper limb muscles [8,9,15]. This is especially important when considering the high prevalence of shoulder injuries in comparison to other workplace and overuse injuries [4,5]. Future work in which direct comparisons between upper limb proximal and distal muscles are performed is a crucial next step in understanding how learning transfer is represented in the upper limb. Furthermore, understanding the relationships that exist between differing musculature groups and neural activity as measured by SEPs is critical as it can lead to outcome predictions for motor function following injury or in recovery for stroke patients—for example, evoked potential amplitudes and latencies measured three weeks post-stroke have been shown to be associated with motor ability measured 10 weeks later [44]. 

Future studies using this same task should look to assess participant perception of task difficulty. Based upon the principle of action-specific perception, participants will view their environment in relation to their ability to successfully perform within that environment [45]. For example, participants who are performing well on the tracing task could perceive the trace to be moving slower or perceive the amplitude and frequency to be smaller. Based upon this hypothesis, how well the participant performs on the task will impact their perception of task difficulty, which could lead to further errors for participants who are not as good at the task to start with, as they become stressed about task performance.

While the use of single electrode EEG recordings has proved sufficient for recording SEP changes in small hand muscles, the results of this study indicate the need for whole-head EEG recordings to assess whole-arm tasks. Certain SEP peaks, such as the frontal N30 peak have multiple neural generators, using a larger number of electrodes would allow for the source localization of these peaks, allowing for a greater discrimination of discrete neural activations during specific phases of learning.

This study collected data for the N18 peak from the frontal contralateral site (Rossi site), as previously done by a number of papers [8,9,10,19,25,40,46]. However, additional studies have shown that recording the N18 peak from an ipsilateral electrode results in decreased contamination of the N18 peak by the succeeding N20 and N24 peaks [31,47]. Future research should consider collection of the N18 peak from an ipsilateral electrode to ensure greater reliability.

While this study occluded the participant’s view of their hand to assess movement-based motor learning and to control for the effects of movement observation, future research should assess task performance with the hand in full view, to further study the generalizability of the task to workplace settings. Additionally, the use of a brace would further ensure the isolation of glenohumeral movement, although this may not be fully reflective of potential movements that are performed in the workplace which may sometimes lack the use of bracing or support for isolation. Future research would benefit from an additional later retention date, to assess the permanence of any neurological changes or performance improvements. In this study, retention was assessed a minimum of 24 h and a maximum of 48 h following acquisition, dependent upon participant availability. The use of a 24–48 h retention period was selected based upon previous research that indicates that 24 h is a sufficient period of consolidation to allow for the investigation of short-term retention effects of motor acquisition paradigms [47,48]. An additional later retention date would further support these results, which indicate that a single session of motor training is sufficient to induce relatively permanent improvements in motor performance. 

## 5. Conclusions

The results of this study indicate that short-latency median nerve SEPs have the capability to reflect changes in cortical and subcortical activity levels in response to novel motor skill acquisition using the proximal upper-limb muscles. This suggests that SEPs can be used as a neural marker of changes in sensorimotor integration following motor acquisition with the entire upper limb. 

In contrast to previous studies completed using the thumb as the prime mover, this study displayed opposite directional amplitude changes in both the N13 and N24 peaks [8,9]. Significant decreases in the spinal N13 SEP peak within the present study suggest differing neural activations within the spinal cord for the proximal upper-limb muscles. In addition, large increases in the N24 peak seen with the proximal upper limb further support previous research suggesting the role of the cerebellum in early motor learning, potentially indicating an increase in cerebellar inhibition during motor learning with the proximal upper limb [10,37,38]. 

## Figures and Tables

**Table 1:** Male and female task accuracy measured as percentage of error. One dot width from the target trace was considered 100% error.

Male				Female		
Subject	Baseline	Post-Acquisition	Retention	Baseline	Post-Acquisition	Retention
**1**	68.86	58.72	51.16	137.88	81.17	73.31
**2**	110.67	58.32	66.67	104.73	63.06	59.78
**3**	79.86	65.30	57.81	104.53	76.47	59.75
**4**	84.14	79.15	60.69	129.06	71.69	76.87
**5**	141.42	64.12	71.40	138.27	81.50	67.54
**6**	92.73	74.87	64.12	99.33	69.14	58.09
**Average**	96.28	66.75	61.98	118.97	73.84	65.89
